# Two Cases With Recurrent Challenges: Unraveling the Complexity of Heyde Syndrome With Rebleeding

**DOI:** 10.7759/cureus.62693

**Published:** 2024-06-19

**Authors:** Tyler J Kingma, Kerry Singh, Shae-Lee Godin, Rachael Hagen, David Bowers

**Affiliations:** 1 Internal Medicine, University of Connecticut, Farmington, USA; 2 Internal Medicine, Hartford Hospital, Hartford, USA

**Keywords:** avms, oral anticoagulation, re-bleeding, aortic stenosis, heyde syndrome

## Abstract

Heyde syndrome is characterized by the association between aortic stenosis and gastrointestinal bleeding. This report examines two cases of Heyde syndrome in elderly females who experience bleeding recurrence within months following aortic valve replacement (AVR). The discussion highlights the controversies surrounding the optimal management of Heyde syndrome, particularly in the context of AVR type (surgical vs. transcatheter) and postoperative complications. The report underscores the need for a multidisciplinary approach to Heyde syndrome management and the importance of individualized treatment strategies considering patient-specific factors such as lesion location and postoperative complications.

## Introduction

Heyde syndrome was first introduced in 1958 as an association between aortic stenosis (AS) and gastrointestinal bleeding (GIB). It was not until the late 1980s that the association of acquired von Willebrand Syndrome (AvWS) was proposed as the mechanism of coagulopathy [1]. Aortic stenosis is a common valvular lesion present in about 5% of the population at age 65 with rates increasing with age. This statistic may be under-documented due to a rising number of aging individuals with undiagnosed mild AS [2]. The prevalence of aortic stenosis in patients with bleeding angiodysplasia is difficult to assess but in limited studies, it has been shown to be between ~3% and 31%. Angiodysplasias are the second most common cause of GIB in the elderly and are associated with longer hospital admissions and increased mortality [3].

## Case presentation

Case 1

**Figure 1:**
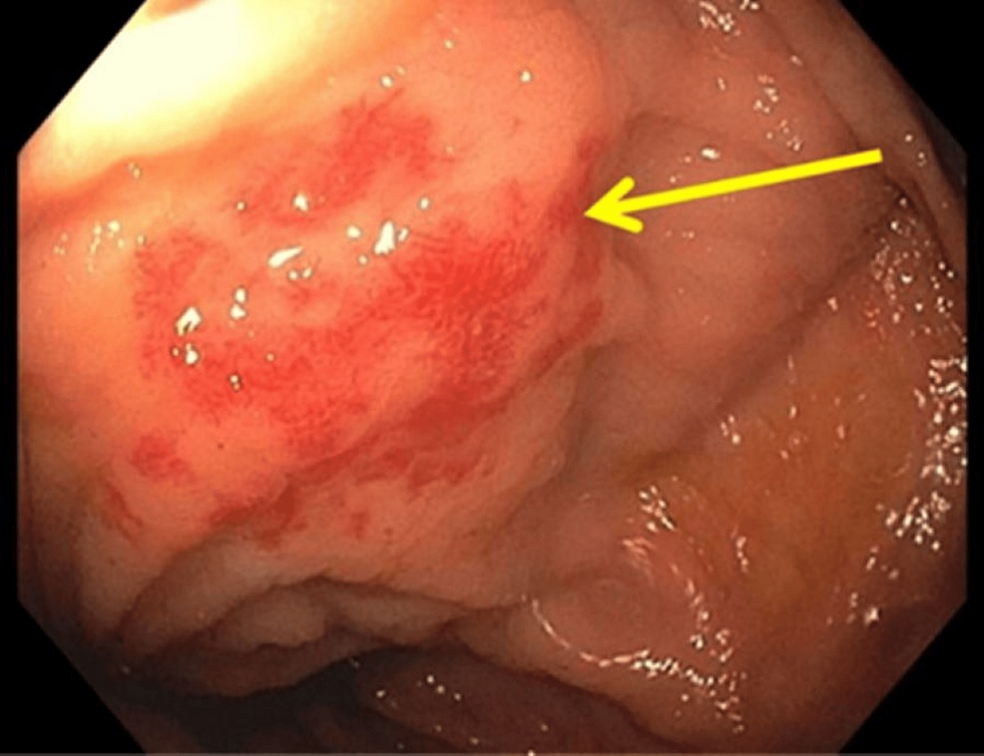
Colonoscopy showing colonic arteriovenous malformation (AVM)

Patient 1 is a 75-year-old female with a past medical history of duodenal, small bowel, and colonic arteriovenous malformations (AVMs), hypertension, and severe aortic stenosis (valve area 0.8 cm^2^ with a peak velocity of 4.1 m/s) status post recent bioprosthetic surgical aortic valve replacement (SAVR) complicated by postoperative paroxysmal atrial fibrillation. She presented to the emergency department with increasing shortness of breath and melanotic stools. She was normotensive, not tachycardic, and required no supplemental oxygen. Physical exam was significant for pallor but otherwise negative. Laboratory workup was significant for a hemoglobin of 6.5 g/dL. At this time, her home apixaban and aspirin were held, and she was transfused one unit packed red blood cells with appropriate hemoglobin response to 7.5 g/dL. Colonoscopy and push enteroscopy showed multiple colonic (Figure 1) and duodenal AVMs that were treated with argon plasma coagulation (APC). Following the procedures, the patient was able to be restarted on 2.5 mg apixaban twice daily while maintaining stability in hemoglobin and without evidence of rebleeding.

Case 2

**Figure 2:**
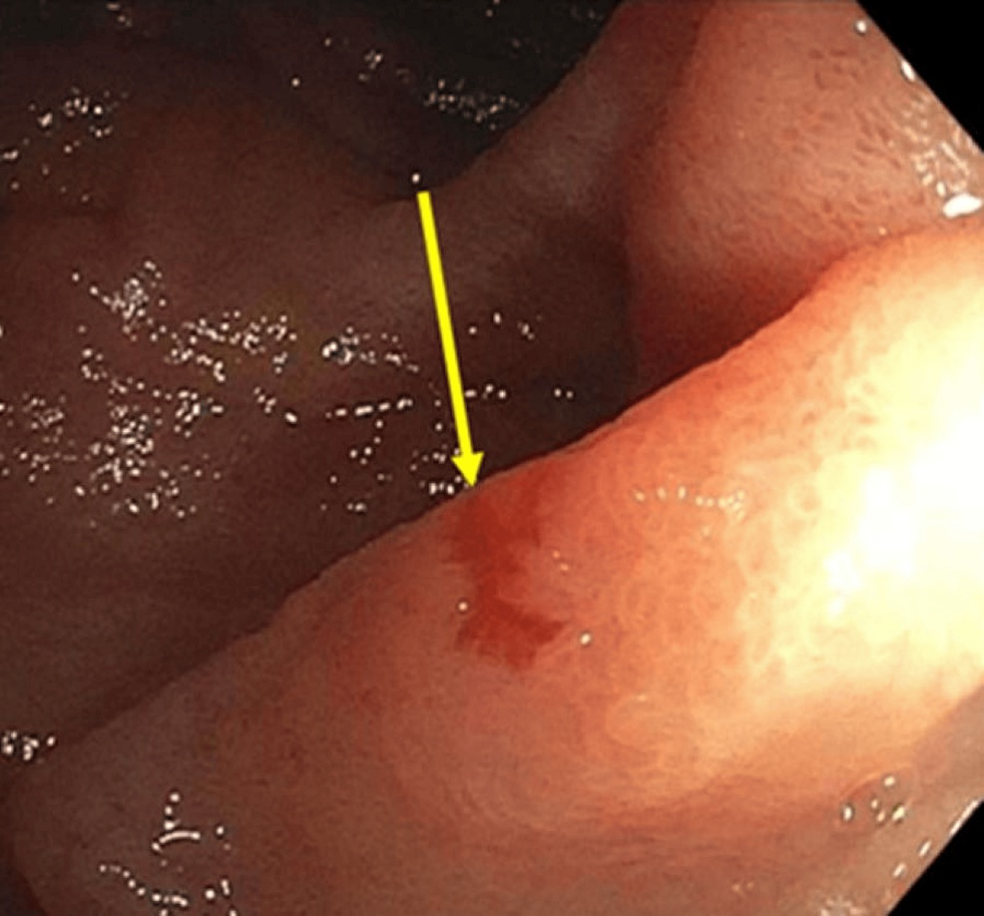
Push enteroscopy with a single, non-bleeding duodenal arteriovenous malformation

Patient 2 is a 79-year-old female with a past medical history of chronic obstructive pulmonary disease on baseline 3 liters nasal cannula, peripheral vascular disease, heart failure with preserved ejection fraction (EF 62%), coronary artery disease, previous GIB secondary to gastric, small bowel, and colonic AVMs and severe aortic stenosis (valve area 0.7 cm^2^ with a peak velocity of 4.7 m/s) status post recent bioprosthetic transcatheter aortic valve replacement (TAVR). She presented to the emergency department after a mechanical fall secondary to increased weakness. Vital signs were stable. Initial laboratory workup was significant for hemoccult positive stool, hemoglobin of 7.9 g/dL, prothrombin time of 13.9 seconds, and potassium of 6.3 mmol/L. After receiving insulin and dextrose, labs the following day showed improvement in potassium to 5.1 mmol/L but down-trending hemoglobin to 6.6 g/dL. One unit of packed red blood cells was transfused with repeat hemoglobin of 8.5 g/dL. Push enteroscopy revealed a single non-bleeding AVM (Figure 2) in the duodenum that was treated with APC. The colonoscopy was aborted due to incomplete colonic preparation. Following the procedures, she was able to be restarted on home aspirin with stabilization in hemoglobin and no further evidence of bleeding.

## Discussion

Contemporary literature suggests that AvWS is the primary mechanism of Heyde syndrome in the setting of severe aortic stenosis. In the context of Heyde syndrome, AVR is the gold standard treatment for the triumvirate of severe aortic stenosis, GIB, and AvWS [4]. In a systematic review of case reports of Heyde syndrome, the average age of patients was noted to be 74.3 ± 9.3 years with a female preponderance [5]. This aligns with the demographics of both our patients. Patients 1 and 2 both underwent bioprosthetic AVR, which has been demonstrated in the literature to be superior to mechanical AVR with regards to rebleeding (15% vs 50% risk) [6]. However, despite undergoing bioprosthetic replacement, our patients suffered rebleeding within several months of their respective procedures. The patients required push enteroscopy for detection of the culprit lesions, which were discovered to be duodenal and colonic for patient 1 and duodenal for patient 2. Indeed, according to the findings of Saha et al., push enteroscopy is superior at the detection of culprit lesions in Heyde syndrome versus colonoscopy and esophagogastroduodenoscopy [7]. Furthermore, angiodysplasia associated with Heyde syndrome typically involves the right colon, with the duodenum accounting for only 14.4% of GIB [5]. In terms of the outcomes of SAVR vs. TAVR, the latter has been demonstrated to have lower rebleeding rates (10.5% vs. 5.7%, respectively) [3]. This is, however, discordant with the findings of both Goldstein and Saha et al., with both papers reporting increased GIB cessation rates with SAVR [7,8]. While Heyde syndrome is already an intrinsically uncommon pathology, determination of the risk factors associated with rebleeding following AVR has proven challenging. One study suggests that the location of intestinal angiodysplasia is the main risk factor for rebleeding following AVR [4]. Like our patients, right colonic and duodenal angiodysplasia was associated with GIB recurrence. Other papers report that mild/moderate postoperative paravalvular leakage (PVL) was associated with rebleeding events [5]. Mechanistically, this seems logical as the shear forces imposed by PVL may contribute to the recurrence of AvWS. The literature also demonstrated that a greater proportion of patients had rebleeding events while on dual antiplatelet therapy than on an antiplatelet and anticoagulant although the data were not statistically significant [5].

## Conclusions

This case series highlights the complexity of managing Heyde syndrome, particularly in elderly patients with significant comorbidities. Despite advancements in aortic valve replacement (AVR) techniques, both surgical (SAVR) and transcatheter (TAVR), recurrent gastrointestinal bleeding (GIB) remains a significant challenge post-procedure. The cases presented underscore the necessity for a multidisciplinary approach to tailor individualized treatment plans.
